# 3-Year outcomes after left atrial appendage closure in patients with nonvalvular atrial fibrillation: cardiomyopathy related with increased death and stroke rate

**DOI:** 10.1186/s12872-023-03054-9

**Published:** 2023-01-17

**Authors:** Chunyu Chen, Yuyi Chen, Lulu Qu, Xi Su, Yanhong Chen

**Affiliations:** 1 Department of Cardiology, Wuhan Asia Heart Hospital, 753Rd Jinghan Road, Wuhan, 430022 Hubei China; 2 Department of Cardiology, The Second Affiliated Hospital of Wuhan University, Zhongnan Hospital, Wuhan, 430060 China

**Keywords:** Left atrial appendage closure, Device-related thrombus, Non-valvular atrial fibrillation, Stroke, Death

## Abstract

**Introduction:**

Left atrial appendage closure (LAAC) is a novel treatment for stroke prevention in high-risk patients with non-valvular atrial fibrillation (NVAF). However, the long-term outcomes after LAAC in Chinese NVAF patients are still lacking.

**Methods:**

This was a single-center, bidirectional, nonrandomized registered study. Patients who underwent LAAC implantation from May 2014 to April 2021 in a large Chinese center were enrolled. The primary endpoint was combined all-cause death and stroke.

**Results:**

From May 2014 to April 2021, a total of 673 NVAF patients were enrolled. The overall successful implantation rate was 97.62% (657 of 673). The rate of perioperative adverse events was 1.19% (8 of 673), including 3 cardiac tamponades, 2 ischemic strokes, one device-related thrombus (DRT) and 2 device dislocations. 604 (92.24%) patients completed the follow-up, the median follow-up period was 36.9 months (IQR 24.8–56.5 months). 16 stroke events occurred in 15 patients (one patient suffered from both hemorrhagic and ischemic strokes). 13 patients (2.15%) had ischemic stroke, and the fatal rate was 0.33% (2 of 604). 3 patients (0.15%) suffered from hemorrhagic stroke, and the fatal rate was 0.17% (1 of 604). The overall stroke rate was 0.74% per-year. The combined death and stroke rate was 1.93% per-year. In the multivariate Cox regression analysis, age ≥ 75 (hazard ratio 2.264, 95% CI 1.074–4.772, *P* = 0.032) and ventricular cardiomyopathy (hazard ratio 2.738, 95% CI 1.060–7.071, *P* = 0.037) were independent predictors of combined mortality and stroke.

**Conclusion:**

The overall successful implantation rate of LAAC was 97.62% and the rate of perioperative adverse events was 1.19% in this study, and the stroke rate was 0.74% per year during the long-term follow-up. Age ≥ 75 years and ventricular cardiomyopathy were independent predictors of the primary endpoint.

*Trial registration* This study was retrospectively registered.

## Introduction

Non-valvular Atrial fibrillation (NVAF) is a common type of cardiac arrhythmia, related with an increased risk of systemic thromboembolism [1, 2]. Previous data has demonstrated that 90% originated from the left atrial appendage (LAA) in NVAF patients [3].

Traditionally, warfarin was a primary treatment protocol of stroke prevention in AF patients. However, it has some disadvantages, such as, high-risk to bleed, susceptible to food and drugs interactions, narrow range of effective doses and frequent need of INR testing. The novel oral anti-coagulants(NOACs) are recommended by most AF guidelines as the first-line stroke prevention medications due to its safety and efficacy [4]. LAA closure (LAAC) has emerged as an alternative for NVAF patients with contraindication for long-term OACs or a high propensity for bleeding or poor drug compliance [5]. The CAP and CAP2 registries [6] showed that LAAC is a safe and effective therapy for stroke prevention in high-risk NVAF patients. The five years meta-analysis of PROTECT and PREVAIL [7] showed that LAAC was non-inferior to warfarin. In long-term follow-up of PRAGUE-17 [8], LAAC remains non-inferior to DOACs in high-risk NVAF patients.

The long-term outcome of LAAC in Chinese AF patients are still lacking. Our bidirectional (prospective, retrospective), single-center study was designed to investigate the long-term outcomes after percutaneous LAAC in patients with NVAF in a registry in mainland China. Periprocedural success and complications were also collected.

## Methods

### Study population

**Fig. 1:**
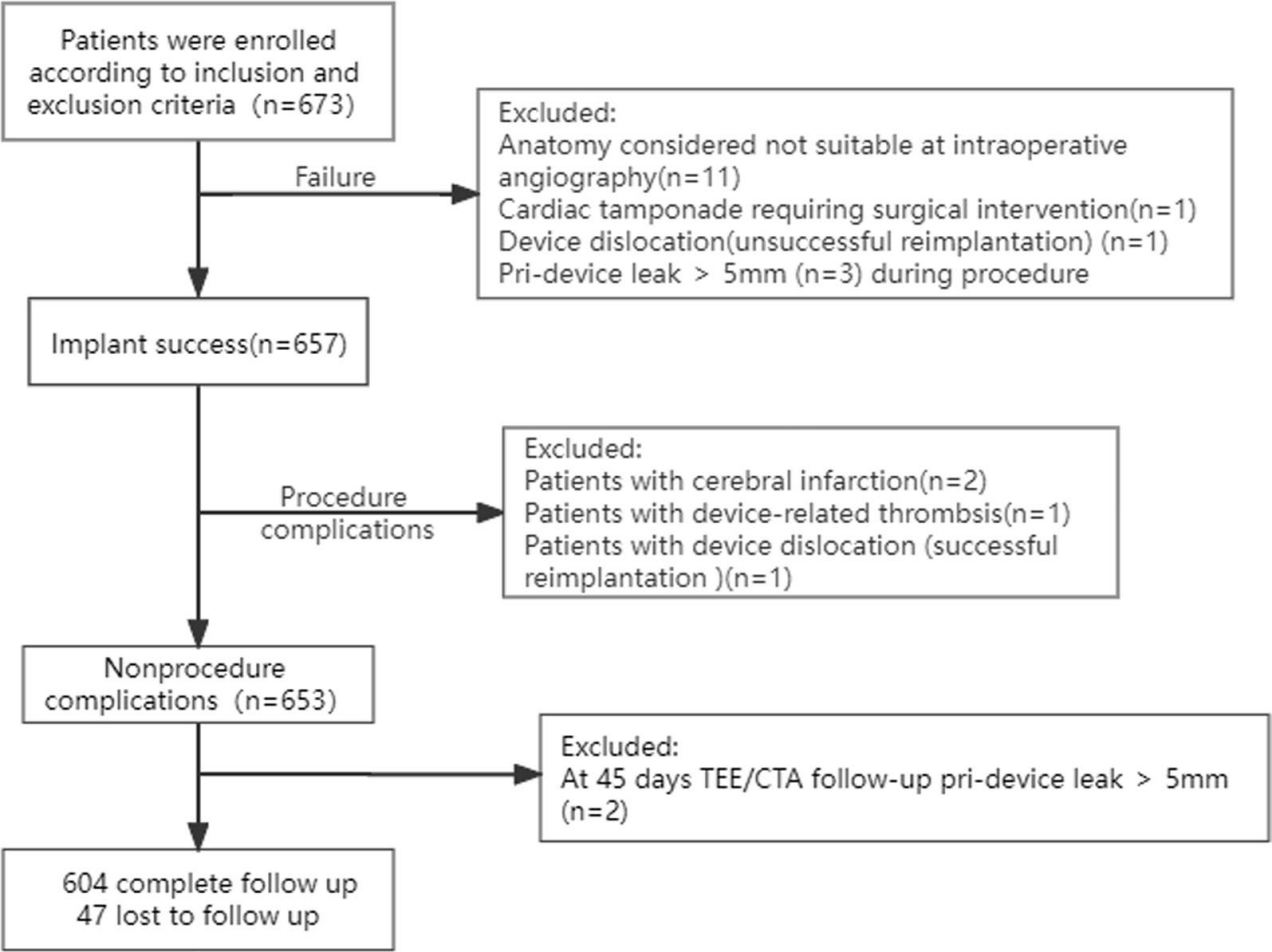
Flow chart of patients who received LAAC devices and completed long-term follow-up

From May 2014 to April 2021, a total of 673 AF patients underwent LAAC were included in our study (retrospectively from 2014 to 2018, prospectively from 2019 to 2021), as shown in Fig. 1. The indications for LAAC operations were as follows: over 18 years of age, presented with paroxysmal or persistent non-valvular AF, CHA2DS2-VASc score ≥ 1, complicated with at least one of the following situations: a high bleeding risk (HAS-BLED score ≥ 3), a contraindication or unwillingness to long-term OACs, or having stroke/TIA despite of regular anticoagulant therapy.

There were exclusion criteria as follows: patients with rheumatic valvular heart disease, mechanical valve replacement, or atrial fibrillation caused by congenital heart disease. The expected lifetime was less than 1 year. Women who are pregnant, lactating or who have a pregnancy plan in the next two years. According to the investigator's judgment, the patients may fail the follow-up.

Baseline clinical characteristics like gender, age, hypertension, diabetes mellitus, coronary artery disease, cardiomyopathy, congenital heart disease, congestive heart failure, ischemic stroke/TIA history, CHA2DS2-VASc score, HAS-BLED score and body mass index (BMI) were recorded for every patient.

The study was approved by the local ethics board. The retrospective data were collected and analyzed anonymously. Informed consent were obtained for all prospectively enrolled patients before the LAAC procedure.

### Device implantation operations

The LAAC device was implanted via a septal approach using a catheter-based delivery system. Briefly, the operations were conducted under general anesthesia and tracheal intubation. After the TEE-guided atrial septum puncture, LAA angiography of the right anterior oblique (RAO)at 30° plus caudal (CAU)at 20° was performed for LAA measurements. LAAC devices with suitable diameters were delivered through a catheter-based delivery system and expanded to close the LAA openings. During the procedures, TEE was performed to confirm the LAA closure. Compression ratio of LAAC devices was calculated immediately after the procedures.

### In-hospital management and follow-up

After the implantation procedures, patients were transferred to the cardiac care unit (CCU) for anesthesia recovery. A trans-thoracic echo-cardiograph (TTE) was performed at the day of the operation to rule out cardiac effusion or device-related embolism. Then, 4–5 days of observing were completed before the patients discharging.

After the device implantation, patients received warfarin (INR ranges of 2.0–3.0) or NOACs for at least 45 days. 45 days after the procedures, TEE or enhanced left atrial CTA was performed to assess the residual flow, stability of the device, and device-related thrombus-formation. After the closure of LAAs was confirmed, anticoagulants (warfarin/NOACs) were discontinued. Patients then took a combination of Aspirin and Clopidogrel for an additional 4.5 months. After that, patients were prescribed with long-term Aspirin alone.

### Definition of endpoints

The primary endpoints of the study were death and stroke rate during the long-term follow-up. The secondary endpoints included TIA, bleeding events and heart failure readmissions during the follow-up. Peri-procedural complications including pericardial tamponade, major-leakage (> 5 mm), device dislocation and device thrombo-embosis were also collected.

### Statistical analysis

Data were presented with mean and standard deviation (SD) for continuous variables or with n and percentage for categorical variables. The Kaplan–Meier graph was used to illustrate the long-term cumulative survival rates during the long-term follow-up, computed with Graphpad Prism 9.0 software. Predictors for death and stroke rate were identified by univariate and multivariable Cox regression analysis. Variables with a *P* < 0.1 in the univariate analysis were incorporated into the multivariable model. All tests were two-tailed, *P* values of less than 0.05 was considered statistically significant. Statistical analyses were completed with SPSS v.26.0 statistical analysis software packet.

## Results

### Baseline characteristics

During the period from May 2014 to April 2021, 673 patients who underwent LAAC in our center were included in this study. Of these, 397 (59.0%) were male, and the average age was 66.00 ± 8.9 years. The mean BMI was 26.04 ± 3.75 kg/m^2^. 5.3% had Ventricular cardiomyopathy, and 32.8% had prior stroke/TIA. 339(50.4%) patients had hypertension, 207 (30.8%) patients had coronary heart disease, and 85 (12.6%) had diabetes mellitus. The average CHA2DS2-VASc score was 2.92 ± 1.67, and the average HAS-BLED score was 1.54 ± 1.08 (Table 1).

**Table 1:** Baseline clinical characteristics

Variable	n = 673
Age (years)	66.0 ± 8.9
Male (n,%)	397 (59.0%)
BMI (kg/m^2^)	26.04 ± 3.75
Persistent AF (n,%)	662 (98.4%)
Clinical history	
Heart failure (n,%)	95 (14.1%)
Hypertension (n,%)	339 (50.4%)
Diabetes mellitus (n,%)	85 (12.6%)
Prior stroke/TIA (n,%)	221 (32.8%)
Coronary artery disease (n,%)	207 (30.8%)
Ventricular Cardiomyopathy (n,%) (n,%)	36 (5.3%)
DCM	21 (3.1%)
HCM	15 (2.2%)
CHA2DS2-VASc score	2.92 ± 1.67
HAS-BLED score	1.54 ± 1.08
Echocardiography	
LAD (mm)	48.80 ± 5.91
LVEDD (mm)	49.20 ± 5.24
LVEF (%)	52.85 ± 5.14

BMI: Body mass index; DCM: Dilated cardiomyopathy; HCM: Hypertrophic cardiomyopathy; LAD: left atrial diameter; LVEDD: left ventricular end-diastolic diameter; LVEF: left ventricular ejection fraction

### Periprocedural complications

Of the 673 patients who underwent LAAC in our study. the implantation attempts were aborted in 11 patients due to unfavorable LAA anatomy (determined by in-procedural angiography). 3 implantation operations were considered unsuccessful due to major peri-device leakages(> 5 mm). Other failure reasons included one device dislocation (the device was successfully snared but re-implantation was not attempted) and one cardiac tamponade requiring surgical intervention and LAA ligation. Ultimately, the successful rate was 97.62% (657 of 673). Of these, 533 (80.76%) were implanted with Watchman devices, 62 (9.38%) were implanted with Lambre devices, 52 (7.88%) were implanted with ACP devices, 12 were implanted with devices of other brands and 1 with Lefort device (Table 2).

**Table 2:** Procedural details

Variable	**n = 673**
Implantation success	657 (97.62%)
Device successfully implanted	660
*Device types*	
Watchman TM	533 (80.76%)
Lambre	62 (9.38%)
ACP	52 (7.88%)
Lefort	1 (0.16%)
Other	12 (1.82%)
*LAA features*	
**TEE diameter (mm)**	21.23 ± 3.86
0°	20.78 ± 3.99
45°	20.43 ± 3.63
90°	21.21 ± 3.95
135°	22.54 ± 3.95
**TEE depth (mm)**	27.30 ± 5.87
0°	27.33 ± 6.37
45°	27.59 ± 5.99
90°	27.85 ± 5.55
135°	27.18 ± 5.53
*Procedure complications*	
Minor	
Pericardial effusion(> 10 mm)(no intervention required)	10 (1.50%)
Major	
Pericardial tamponade (percutaneous peri-cardium drained or surgically repaired)	3 (0.45%)
Ischemic stroke	2 (0.30%)
Device dislocation	2 (0.30%)
Device-related thrombo-embolization	1 (0.15%)

TEE: trans-esophageal echocardiography; ACP: Amplatzer Cardia Plug

The rate of peri-operative adverse events was 1.19% (8 of 673). Pericardial tamponade occurred in 3 patients (one patient had surgical LAA ligation, the other 2 underwent percutaneous pericardial drainage). Peri-procedural cerebral infarction was recorded in 2 patients. One received non-interventional medical treatment and fully recovered. Another one had hemiplegia and suffered from a second stroke 2 months after LAAC, which caused cognitive impairment and worsened limb hemiplegia. Device dislocation occurred in 2 patients. One was successful snared but re-implantation was not attempted. The patient had repeated ischemic stroke attacks (three times) during the long-term follow-up, leaving cognitive impairment and limb hemiplegia. For the other patient, the dislocated LAAC device was successfully snared and a new device was implanted. He completed the follow-up without any events.

Device-related thrombus (DRT) occurred in 1 patient (0.15%). The patient was put on anticoagulation treatment and no cerebrovascular event occurred during the peri-procedure period. However, the thrombosis on the device did not disappear at the 45 days TEE follow-up. Despite of the long-term anticoagulation treatment, the patient had fatal ischemic stroke 4 years after LAAC despite of continued anticoagulation. Pericardial effusion of more than 10 mm occurred in 10 patients (1.49%) all of which had no symptoms and absorbed simultaneously.

### Clinical outcomes during follow-up

At the 45-days follow-up, 575 patients received TEE or left atrial CTA. Major leakage or DRT occurred in 13 patients (Table 3). All 13 patients continued anticoagulation and completed the follow-up. 5 patients had major leakage (> 5 mm) around the devices. of whom 3 patients already had major leakage during the procedures. DRT was observed in 8 patients.

**Table 3:** Outcome of TEE or left atrial CTA follow-up

Variable	n = 575
Follow-up of TEE or left auricle CTA (n,%)	575 (87.65%)
Peri-device leakage, (n,%)	239 (41.57%)
Major leakage(> 5 mm) (n,%)	5 (0.87%)
Minor leakage(≤ 5 mm)(n,%)	234 (40.70%)
Mean leakage size (mm)	2.52 ± 1.78
Device thrombosis (n,%)	8 (1.39%)

**Fig. 2:**
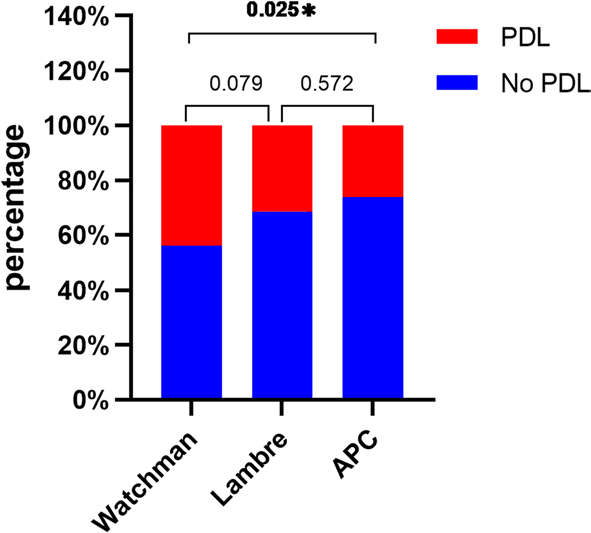
The rate of PDLs among the three device types during the 45-day follow-up

The most frequent types implanted were Watchman (80.76%), Lambre (9.38%) and ACP (7.88%). At the 45-days follow-up, the leakage rate was 43% (205/466) for the Watchman devices, 31% (17/54) for ACP devices, and 26% (11/42) for the Lambre devices. The differences were statistically significant (*P* < 0.05) (Fig. 2). The rate of peri-procedural complications and the rate of DRTs were not different among the three device types.

During a median follow-up of 36.9 months (2018 patient-years). One of the patients with major leakage had ischemic stroke 2 years after the procedure, leaving cognitive impairment and limb hemiplegia. Among the 8 patients with DRT, 2 patients lost follow-up, and 6 patients had no events during the long-term follow-up.

After the exclusion of 47 patients who were lost to follow-up and 22 patients with periprocedural complications or major leakage, complete data were obtained in 604 patients (Fig. 1). The loss rate was 7.76%. The overall death rate was 4.47% (27 of 604). The cardiac death rate was 1.66%(10 of 604), including heart failure 0.99% (6 of 604), and myocardial infarction 0.66% (4 of 604) (Table 4).

**Table 4:** Clinical outcomes during long-term follow-up

Variable	n = 604
All-cause death	27 (4.47%)
Heart failure	6 (0.99%)
Ischemic stroke	2 (0.33%)
Acute myocardial infarction	4 (0.66%)
Cerebral hemorrhage	1 (0.17%)
Unexplained death	14 (2.32%)
All-cause stroke	15 (2.48%)
Ischemic stroke	13 (2.15%)
Fatal stroke	2 (0.33%)
Disabling stroke	6 (0.99%)
Nondisabling stroke	5 (0.83%)
Hemorrhagic stroke	3 (0.51%)
Fatal stroke	1 (0.17%)
Disabling stroke	1 (0.17%)
Nondisabling stroke	1 (0.17%)
TIA	10 (1.66%)
All-cause bleeding	64 (10.60%)
Cerebral bleeding	3 (0.50%)
Non-major bleeding	61 (10.10%)
Heart failure readmission	58 (9.60%)

TIA: transient ischemic attacks

The overall stroke rate was 0.74% per-year. All-cause stroke occurred in 15 patients, including 13 ischemic stroke (2.15%) and 3 hemorrhagic stroke (one patient had one ischemic stroke and one hemorrhagic stroke). The fatality rate of ischemic stroke was 0.33% (2 of 604). The disabling rate of ischemic stroke was 0.99% (6 of 604). The fatality rate of hemorrhagic stroke was 0.17% (1 of 604). The disabling rate of hemorrhagic stroke was 0.17% (1 of 604). The cumulative rate of TIA was 1.66% (10 of 604).

**Fig. 3:**
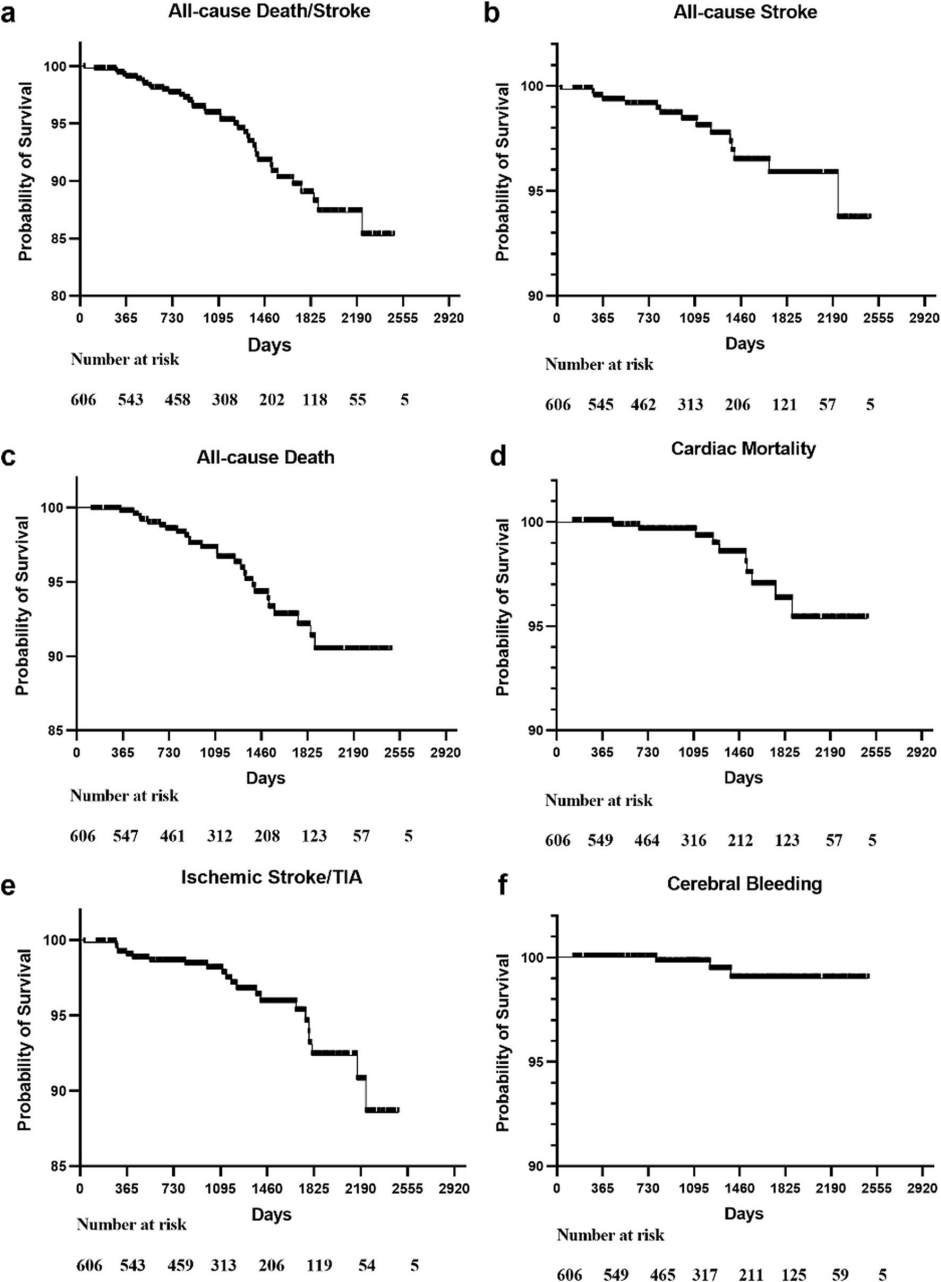
**a** Kaplan–Meier graph showing long-term cumulative survival from all-cause death and stroke; **b** Kaplan–Meier graph showing long-term cumulative survival from all-cause stroke; **c** Kaplan–Meier graph showing long-term cumulative survival from all-cause death; **d** Kaplan–Meier graph showing long-term cumulative survival from cardiac mortality; **e** Kaplan–Meier graph showing long-term cumulative survival from ischemic stroke and TIA; **f** Kaplan–Meier graph showing long-term cumulative survival from cerebral bleeding

Bleeding occurred in 64 patients, including cerebral bleeding in 3 patients, and minor bleeding in 61 patients (e.g., gastrointestinal bleeding, gingival bleeding, tarry stool, nasal hemorrhage, and all minor bleeding improved after discontinuing antiplatelet or anticoagulant drugs). Heart failure readmission occurred in 58 patients. The Kaplan–Meier graph was used to illustrate the complications on long-term cumulative survival **(**Fig. 3).

**Fig. 4:**
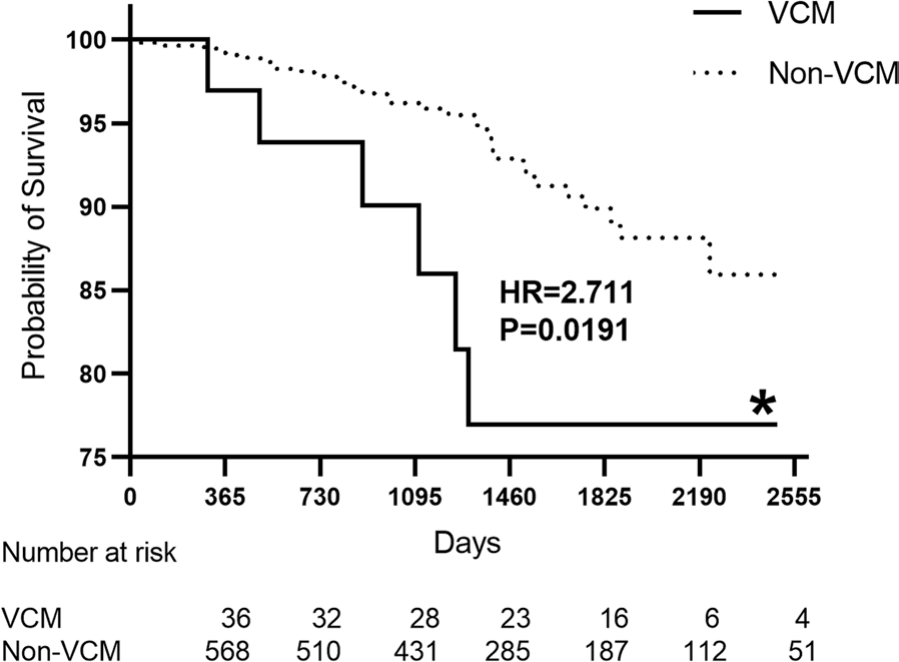
Kaplan–Meier graph showing long-term cumulative survival according to the presence and absence of ventricular cardiomyopathy (VCM)

The cumulative rate of combined death and stroke was 1.93% per-year. In the univariate Cox regression analysis (Table 5), the history of cardiomyopathy (hazard ratio 2.715, 95% CI 1.137–6.484, *P* = 0.025) was a predictor of mortality and stroke after LAAC implantation. In the multivariate Cox regression analysis, age ≥ 75 (hazard ratio 2.074, 95% CI 1.003–4.285, *P* = 0.049) and the history of cardiomyopathy (hazard ratio 2.959, 95% CI 1.231–7.116, *P* = 0.015) were independent predictors of mortality and stroke after LAAC implantation at long-term follow-up (Fig. 4).

**Table 5:** Predictors of all-cause mortality/stroke at long-term follow-up with multivariate Cox regression analysis

Variable	Univariate		Multivariable	
	HR (95% CI)	*P*	HR (95% CI)	*P*
Age ≥ 75(years)	1.926 (0.937–3.958)	0.074	**2.074 (1.003–4.285)**	0.049*
Male sex	1.594 (0.807–3.148)	0.179	–	
Hypertension	0.740 (0.392–1.396)	0.353	–	
History of ischemic stroke	1.230 (0.652–2.322)	0.522	–	
Coronary artery disease	1.004 (0.515–1.954)	0.992	–	
History of heart failure	1.765 (0.893–3.487)	0.102	–	
Ventricular cardiomyopathy	**2.715 (1.137–6.484)**	0.025*	**2.959 (1.231–7.116)**	0.015*
Diabetes mellitus	0.821 (0.321–2.099)	0.680	–	

*P < 0.05 are shown in bold

Cl: confidence interval; HR: hazard ratio

## Discussion

The main findings of this study are: (1) LAAC is a safe and effective stroke prevention treatment with satisfactory outcomes in Chinese NVAF population; (2) The rate of combined death and stroke was 1.93% per-year; (3) Age ≥ 75 yr and the history of ventricular cardiomyopathy were independent risk factors for combined death and stroke after LAAC. This long-term followed cohort provides more representational clinical outcomes for NVAF patients from main land Chinese population.

LAAC is an alternative to warfarin or NOACS for lifelong stroke prevention in high-risk patients with NVAF [9]. Especially in elderly NVAF patients with high bleeding risk [10]. The ESC Guidelines recommended that LAAC may be considered for stroke prevention in patients with NVAF and contraindications for long-term anticoagulant treatment (class IIb, level of evidence B) [4].

The LAAC success rate increased from 90.9% in PROTECT to 94.4% in CAP, 95.1% in PREVAIL, 94.8% in CAP2, and reached 98.5% in EWOLUTION [11]. The total successful rate was 97.62% in our study, being comparable to that in the EWOLUTION study Among the 11 patients whose implantation were aborted, the most common reason had been unfavorable anatomy (large opening of the LAAC or mismatch between the LAA and the device). So assessment of the LAA anatomy is crucial for success implantation of LAAC devices. Major leakage(> 5 mm) was observed in 3 patients during the procedures, which were also considered unsuccessful. In another 2 failed patients, one had device dislocation and re-implantation was not attempted; the other had cardiac tamponade requiring surgical intervention.

The rate of perioperative adverse events was about 1.19%, significantly lower than MAUDE database [12] and NCDR registry study [13]. DRT is a fatal complication of LAAC, which is associated with the risk of visceral thromboembolism [14]. Aggressive treatment is required once DRT formation is detected. At 45-day follow-up, 575 patients received TEE or left atrial CTA. A total 8 cases of DRT were detected, among whom 2 patients lost follow-up, and 6 patients had no events during the follow-up. In the previous studies, long-term DRT after 45 days was not uncommon. Boersma et al. [15] reported that there was no statistical relations between DRT and types of anticoagulants post LAAC. Chen et al. [16] reported that NOACs after LAAC appear to be as effective as warfarin in preventing DRT, with lower bleeding rate, during a mean follow-up of 868 days. A single-center study [17] investigated 319 patients who underwent LAAC, TEE follow-up was conducted at intra-procedure, 45 days and 6 months after the index procedures. At 6 months after LAAC, DRT was detected in 14 patients and might be related with peri-device leakage. Sedaghat et al. [18] showed that among 835 patients who completed the TEE follow-up, DRT was detected in 4.1% of patients at 54 days (median) and 91.2% within 3 months. Persistent AF was an independent factor of device thrombosis, and new onset DRT was often detected 3 months after LAAC. Other studies have emphasized the need of TEE to detect DRT more than 6 months after LAAC [19]. Unfortunately, we did not perform long-term TEE follow-up in this study, so the rate of new onset DRT after the termination of oral anticoagulation was unclear. Anyway, considering the overall stroke and TIA rate during the follow-up, we might conjecture that the rate of long term DRT is reasonably low, which could be attributed to active and regular anticoagulation and control of AF.

During a median follow-up of 36.9 months (2018 patient-years) in our study. The rate of death and stroke was 1.93% per-year. Meta-analysis outcomes from the PREVAIL and PROTECT AF trials [7] showed that the all-cause stroke and systemic embolism rate was 1.7% per-year, and the all-cause death was 3.6% per-year in the LAA device group. Data from the Boersma study [15] analyzed 1020 patients during a 2-year follow-up, the stroke rate was 1.3% per-year, the mortality rate was 16.4%. During 3.5 years of follow-up (1,354 patient-years), the mortality rate and all-cause stroke rate in the LAAC arm of PRAGUE-17 trial [8] was 6.23% per-year and 2.08% per-year, respectively. All-cause mortality rates in the CAP and CAP 2 registries [6] were 4.27% and 6.24%, respectively. And all-stroke were 1.48% and 2.25%, respectively. Chiu et al. [20] reported that, during mean 28 months follow-up, the ischemic stroke rate was 1.9% per-year in the Watchman group and 1.4% per-year in the ACP/Amulet group. Compared with aforementioned studies, our study showed comparatively lower mortality and stroke rate. which might be attributed to the fact that our patients had been relatively younger and had lower CHA2DS2-VASc score. Besides, patients with major leakage or DRTs in the procedure and at the 45-day TEE follow-up were excluded from the long-term follow-up analysis, as was shown in Fig. 1.

In multivariate Cox regression analysis, age ≥ 75 (hazard ratio 2.074, 95% CI 1.003–4.285, *P* = 0.049) and ventricular cardiomyopathy (hazard ratio 2.959, 95% CI 1.231–7.116, *P* = 0.015) were independent predictors of mortality and stroke after LAAC implantation during the long-term follow-up. Among our ventricular cardiomyopathy patients, 15 are HCM (41.7%). The rate of stroke or death in the HCM patients were particularly high (2 strokes and 2 deaths). While HCM patients are at high risks for stroke and long-term anticoagulation is recommended [21, 22], LAAC does not seem like a good alternative.

One review of HCM management [23] reported that the mortality and stroke rates of HCM patients with AF decreased by 11.6 and 3.5 times, respectively, compared with 2001. For HCM patients with newly-onset symptomatic AF or asymptomatic AF, anticoagulant should be prescribed after evaluating the advantages and disadvantages. Bin-Feng Mo et al. [24] reported that LAAC is safe for primary and secondary stroke prevention in patients with HCM and AF. There were no thromboembolic or death events in HCM patients after LAAC during a mean follow-up of 28.4 months. However, our study showed high stroke and death rate. The patients in our study are predominantly persistent AF, had larger LA and greater LV wall thickness. It seems that in these patients LAAC is not suitable for stroke prevention. Zhang et al. [25] in their study also suggested that LAAC might be unsuitable for AF with ventricular cardiomyopathy because their thrombi were frequently formed in the sites other than LAA.

### Limitations

This study is a single-center, non-randomized study, which lacks a control group and is prone to selective bias. In our study, ventricular cardiomyopathy was independently related with post LAAC death and stroke rate during the long term follow-up. However, the enrolled patients with ventricular cardiomyopathy were mainly non-ischemic cardiomyopathy. Ischemic cardiomyopathy had been very rare and thus was not included in the analysis. Our outcome should be interpreted and extrapolated with caution.

## Conclusion

The overall successful implantation rate of LAAC was 97.62% and the rate of perioperative adverse events was 1.19% in this study, and the stroke rate was 0.74% per year during the long-term follow-up. Age ≥ 75 years and ventricular cardiomyopathy were independent predictors of the primary endpoint.

## Data Availability

The datasets used and analyzed during the current study are available from the corresponding author on reasonable request.

## Abbreviations

LAAC: Left atrial appendage closure
NVAF: Non-valvular atrial fibrillation
DRT: Device-related thrombus
NOACs: Novel oral anti-coagulants
BMI: Body mass index
CCU: Cardiac care unit
TTE: Trans-thoracic echo-cardiograph
TEE: Trans-esophageal echocardiography
TIA: Transient ischemic attacks
SD: Standard deviation
DCM: Dilated cardiomyopathy
HCM: Hypertrophic cardiomyopathy
PDL: Peri-device leakage
LAD: Left atrial diameter
LVEDD: Left ventricular end-diastolic diameter
Cl: Confidence interval
HR: Hazard ratio
LVEF: Left ventricular ejection fraction
